# Protective Effects of Colomast^®^, a New Formulation of Adelmidrol and Sodium Hyaluronate, in a Mouse Model of Acute Restraint Stress

**DOI:** 10.3390/ijms21218136

**Published:** 2020-10-30

**Authors:** Ramona D’Amico, Rosalba Siracusa, Roberta Fusco, Marika Cordaro, Tiziana Genovese, Alessio Filippo Peritore, Enrico Gugliandolo, Rosalia Crupi, Daniela Impellizzeri, Salvatore Cuzzocrea, Rosanna Di Paola

**Affiliations:** 1 Department of Chemical, Biological, Pharmaceutical and Environmental Sciences, University of Messina, Viale Ferdinando Stagno D’Alcontres 31, 98166 Messina, Italy; rdamico@unime.it (R.D.); rsiracusa@unime.it (R.S.); rfusco@unime.it (R.F.); tiziana.genovese@unime.it (T.G.); aperitore@unime.it (A.F.P.); egugliandolo@unime.it (E.G.); dipaolar@unime.it (R.D.P.); 2 Department of Biomedical, Dental and Morphological and Functional Imaging University of Messina, Via Consolare Valeria, 98125 Messina, Italy; cordarom@unime.it; 3 Department of Veterinary Sciences, University of Messina, 98168 Messina, Italy; rcrupi@unime.it; 4 Department of Pharmacological and Physiological Science, Saint Louis University School of Medicine, 1402 South Grand Blvd, St Louis, MO 63104, USA

**Keywords:** Colomast^®^, restraint stress, inflammation, tight junction

## Abstract

Stress is generally defined as a homeostatic disruption from actual or implied threats and alters the homeostatic balance of different body organs, such as gastrointestinal function and the hypothalamic-pituitary-adrenal axis (HPA), inducing the release of glucocorticoid hormones. Stress is also known to be a risk factor for the development of depression and anxiety. However, until today there are no suitable therapies for treating of stress. The aim of this study was to explore the protective effect of Colomast^®^, a new preparation containing Adelmidrol, an enhancer of physiological of palmitoylethanolamide (PEA), and sodium hyaluronate in an animal model of immobilization stress. Acute restraint stress (ARS) was induced in mice by fixation for 2 h of the four extremities with an adhesive tape and Colomast^®^ (20 mg/kg) was administered by oral gavage 30 min before the immobilization. Colomast^®^ pre-treatment was able to decrease histopathological changes in the gastrointestinal tract, cytokines expression, neutrophil infiltration, mast cell activation, oxidative stress, as well as modulate nuclear factor NF-kB and apoptosis pathways after ARS induction. Moreover, Colomast^®^ was able to restore tight junction in both ileum and hippocampus and cortex. Additionally, we demonstrated that Colomast^®^ ameliorated depression and anxiety-related behaviours, and modulate inflammatory and apoptosis pathways also in brain after ARS induction. In conclusion, our results suggest Colomast^®^ to be a potential approach to ARS.

## 1. Introduction

Stress is defined as a state in which homeostasis is actually threatened or perceived to be so, which may challenge an organism’s well-being [1]; homeostasis is re-established by a complex repertoire of behavioral and physiological adaptive responses of the organism [2]. Stress, whether physical or emotional, impairs the physiological/psychological balance of the different body organs and activates the hypothalamic-pituitary-adrenal (HPA) axis and induces the release of glucocorticoid hormones that exert widespread effects [3]. It is well known that, in response to stress, different molecular pathways become activated, including inflammation [4,5], oxidative stress [6,7], as well as apoptotic cell death pathways [8]. These pathways are also considered as major components of the pathophysiology of neurodegenerative disorders, and may predispose the nervous system to the subsequent development of neurodamages during the life when encountering stressor conditions [9,10].

Acute restraint stress (ARS) is an easy and convenient method of inducing both psychological and physical stress, resulting in restricted mobility and aggression. Restraint is painless and does not cause physical harm to the animals, but does activate the HPA-axis and increases the production of glucocorticoids, initiating the deleterious effects of stress [11]. In fact, stress is hypothesized to be one of the triggering factors that causes mental disorders, such as anxiety and depression, but also a risk factor for numerous disorders affecting the gastrointestinal (GI) tract, including changes in gut motility, secretion, and absorption [12,13]. In a recent study, Mazzon and colleagues [14] demonstrated that immobilization stress induced an increase in tight junction (TJ) permeability in the rat terminal ileum. These changes were mainly due to modifications and redistribution of the TJ transmembrane protein occludin and plaque protein zonula occludens-1 (ZO-1).

Given the importance of psychosocial stresses in the modern human lifestyle and the association of stress with various disorders, it is therefore necessary to investigate new therapeutic strategies for treating of ARS.

Adelmidrol, a diethanolamide derivative of natural azelaic acid, is an enhancer of physiological of palmitoylethanolamide (PEA), an endogenous fatty acid amide belonging to ALIAmide family (autacoid local injury antagonist amides). The anti-inflammatory properties of Adelmidrol have been repeatedly demonstrated in natural as well as experimentally-induced acute and chronic inflammation [15,16,17,18,19,20] and might depend—at least in part—to its ability to increase the endogenous concentration of PEA [21]. In combination with sodium hyaluronate, a physiological linear glycosaminoglycan of the extracellular matrix, Adelmidrol was also shown to exert beneficial effects in osteoarthritis [22] as well as spinal cord injury [23]. Here, we investigated the effects of a new formulation consisting of Adelmidrol and sodium hyaluronate, namely Colomast^®^, in an experimental model of ARS.

## 2. Results

### 2.1. In Vivo Adelmidrol Absorption

Considering the sensitivity of LC-MS/MS (limit of quantification (LOQ) = 71.4 ng/mL), serum levels of Adelmidrol in all samples were less than LOQ, suggesting a local action on colon tissue and non-systemic effect (Table 1).

### 2.2. Effect of Colomast^®^ Pre-Treatment on Stress Hormones and Fecal Output

**Figure 1:**
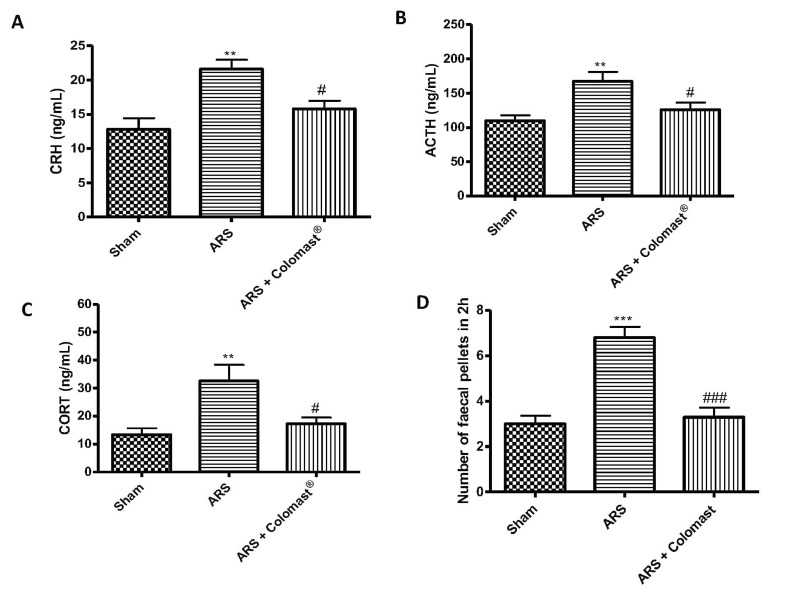
Effect of Colomast^®^ pre-treatment on stress hormones and fecal output. Elisa kit of hypothalamic corticotrophin-releasing hormone (CRH) (**A**), adrenocorticotrophic hormone (ACTH) (**B**), and corticosterone (CORT) (**C**) expressed in plasma. Fecal pellet outputs (**D**). Values shown are means ± SEM of six animals in each group. ** *p* < 0.01 vs. sham; *** *p* < 0.001 vs. sham; # *p* < 0.05 vs. ARS group; ### *p* < 0.001 vs. ARS group.

The levels of hormones involved in stress response—namely hypothalamic corticotrophin-releasing hormone (CRH), adrenocorticotrophic hormone (ACTH), and corticosterone (CORT)—were measured in plasma. Two hours after immobilization, stress hormones levels were significantly increased in ARS mice compared to the sham group, while Colomast^®^ pre-treatment decreased levels of CRH, ACTH, and CORT (Figure 1A–C, respectively).

Additionally, defecation was increased during exposure to stress, as shown by the higher number of fecal pellets expelled by stressed mice compared to the sham group (Figure 1D). The fecal pellet outputs were significantly decreased in the Colomast group compared to the ARS group (Figure 1D).

### 2.3. Effect of Colomast^®^ Pre-Treatment on Tissue Damage

**Figure 2:**
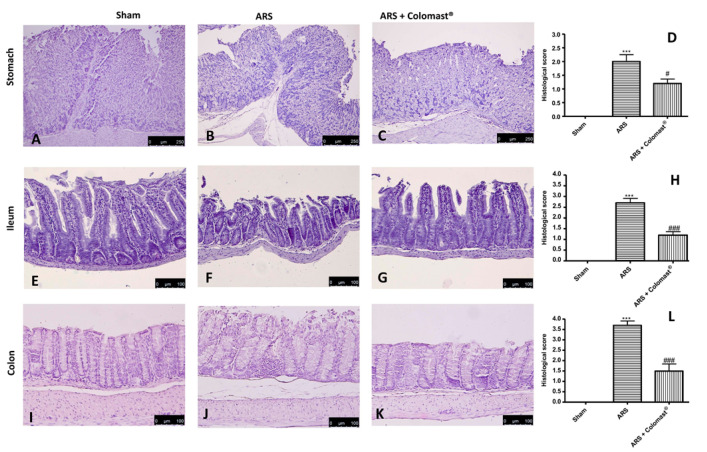
Effect of Colomast^®^ pre-treatment on histological damage. Histological evaluation of stomach: sham (**A**); Acute restraint stress (ARS) (**B**); Colomast^®^ (**C**); histological score (**D**). Histological evaluation of ileum: sham (**E**); ARS (**F**); Colomast^®^ (**G**); histological score (**H**). Histological evaluation of colon: sham (**I**); ARS (**J**); Colomast^®^ (**K**); histological score (**L**). Images are figurative of at least three independent experiments. Values shown are means ± SEM of six animals in each group. For the histology, a 10× magnification is shown (250-µm scale bar) for stomach; a 20× magnification is shown (100-µm scale bar) for ileum and colon. *** *p* < 0.001 vs. sham; # *p* < 0.05 vs. ARS group; ### *p* < 0.001 vs. ARS group.

To evaluate the effect of Colomast^®^ on ARS-induced damage, we evaluated the histopathological changes in the stomach, ileum, and colon. The ARS group presented severe histological injury of the gastric mucosa, characterized by edema, neutrophil infiltration, and morphological changes (Figure 2B,D) compared to the sham group (Figure 2A,D). A severe loss of epithelial cells was observed at the ileum (Figure 2F,H) and colon level (Figure 2J,L) in the ARS group compared to the sham group (Figure 2E,H and Figure 2I,L, respectively). Colomast^®^ (20 mg/kg) pre-treatment significantly reduced inflammatory cell infiltration and protected stomach, ileum, and colon against tissue damage (Figure 2C,G,K and Figure 2D,H,L, respectively).

### 2.4. Effect of Colomast^®^ Pre-Treatment on Mucous Secreting Cells

**Figure 3:**
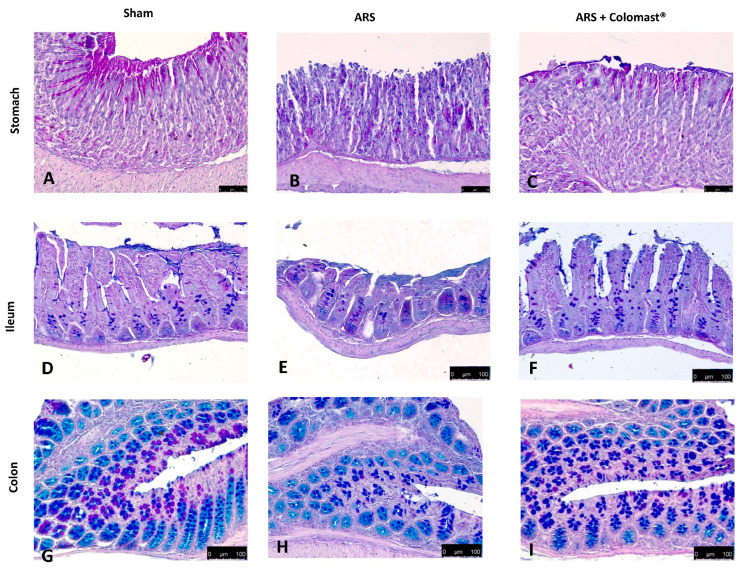
Effect of Colomast^®^ pre-treatment on alteration of mucous secreting cells. Alcian blue/PAS staining in stomach section: sham (**A**), ARS (**B**), Colomast^®^ (**C**); in ileum section: sham (**D**), ARS (**E**), Colomast^®^ (**F**); in colon section: sham (**G**), ARS (**H**), Colomast^®^ (**I**). Images are figurative of at least three independent experiments. Values shown are means ± SEM of six animals in each group. For Alcian blue/PAS staining, a 40× magnification is shown (75-µm scale bar) for stomach; a 20× magnification is shown (100-µm scale bar) for ileum and colon.

Alcian blue/PAS technique was applied to detect mucous secreting cells in stomach, ileum, and colon tissues. In control mice, the mucous secreting cells in the stomach sections deeply stained with Alcian blue/PAS (Figure 3A); in contrast, 2 h after ARS, the sparse mucous secreting cells displayed light staining with Alcian blue/PAS, suggesting gastric mucosa atrophy (Figure 3B). Treatment with Colomast^®^ (20 mg/kg) tended to normalize the staining of mucous secreting cells (Figure 3C). At the ileum (Figure 3E) and colon level (Figure 3H), mucosal changes characterized by acid mucin secretion were observed 2 h post-immobilization compared to sham group (respectively Figure 3D,G). In contrast, sections from the Colomast^®^ group showed decreased acid mucin secretion either at ileum (Figure 3F) and colon (Figure 3I).

### 2.5. Effect of Colomast^®^ Pre-Treatment on Ars-Induced Mast Cell Degranulation in Ileum and Colon

**Figure 4:**
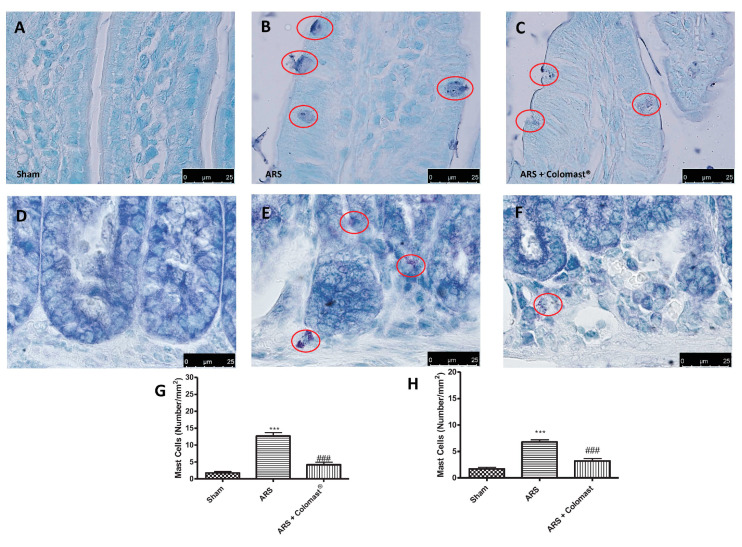
Effect of Colomast^®^ pre-treatment on ARS-induced mast cell degranulation. Evaluation of mast cell degranulation in ileum sections: sham (**A**); ARS (**B**); Colomast^®^ (**C**); mast cells count (**G**). Evaluation of mast cell degranulation in colon sections: sham (**D**); ARS (**E**); Colomast^®^ (**F**); mast cells count (**H**). The red circle indicated mast cells. Images are figurative of at least three independent experiments. Values shown are means ± SEM of six animals in each group. For the mast cells, a 100× magnification is shown (25-µm scale bar). *** *p* < 0.001 vs. sham; ### *p* < 0.001 vs. ARS group.

Since ARS injury causes an important mucosal mast cell activation [24], we evaluated the effect of Colomast^®^ on the number of activated mast cells. Toluidine blue staining highlighted the absence of mast cells in the ileum of sham mice (Figure 4A,G) while degranulating mast cells were increased in the ARS group (Figure 4B,G), with the increase being significantly counteracted by oral pre-treatment with Colomast^®^ (Figure 4C,G). At the same way, Colomast^®^ reduced the number of activated mast cells (Figure 4F,H) in colon sections compared to the ARS group (Figure 4E,H).

### 2.6. Effect of Colomast^®^ Pre-Treatment on Inflammation Pathway

**Figure 5:**
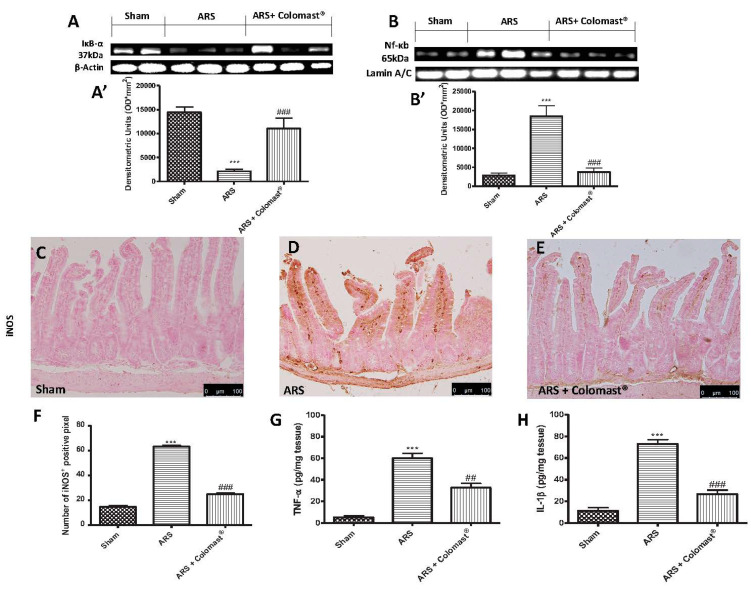
Effect of Colomast^®^ pre-treatment on inflammation pathway. Western blots and respectively densitometric analysis of IkB-α (**A**,**A’**), NF-kB p65 (**B**,**B’**). Immunohistochemistry evaluation of iNOS expression on sham (**C**), ARS (**D**), and Colomast^®^ (**E**); graphical quantification (**F**). Elisa kit of TNF-α (**G**) and IL-1β (**H**) expressed in ileum tissue. A demonstrative blot of lysates with a densitometric analysis for all animals is showed. Images are figurative of at least three independent experiments. Values shown are means ± SEM of six animals in each group. For the immunohistochemistry, a 20× magnification is shown (100-µm scale bar). *** *p* < 0.001 vs. sham; ## *p* < 0.01 vs. ARS group; ### *p* < 0.001 vs. ARS group.

To investigated the cellular mechanisms whereby treatment with Colomast^®^ attenuated the inflammatory process induced by ARS, IκB-α degradation and nuclear translocation of NF-kB p65 were assessed by Western blot analysis. This set of experiments was limited to ileum. The results obtained showed a basal expression of IκB-α in ileum of sham groups (Figure 5A,A’), while the levels of IκB-α significantly decreased in samples collected from vehicle-treated mice (Figure 5A,A’). Colomast^®^ administration reduced degradation of IκB-α induced by ARS (Figure 5A,A’). Vice versa, NF-κB p65 levels in the ileum nuclear fractions were substantially increased after 2 h of immobilization compared to the sham-treated mice (Figure 5B,B’). Treatment with Colomast^®^ (20 mg/kg) reduced the translocation of NF-κB p65 protein (Figure 5B,B’).

In order to determine whether Colomast^®^ may modulate the secretion of pro-inflammatory cytokines, we also analyzed ileum levels of tumor necrosis factor alpha (TNF-α) and interleukin (IL)-1β by ELISA kits. A substantial increase of TNF-α (Figure 5G) and IL-1β (Figure 5H) was found in the ileum tissues collected from ARS-mice, while Colomast^®^ pre-treatment at dose of 20 mg/kg reduced in a significant manner the levels of TNF-α (Figure 5G) and IL-1β (Figure 5H). Additionally, we also analyzed iNOS expression by immunohistochemistry analysis of ileum sections. No positive staining for iNOS was observed in ileum tissues of the sham-treated group (Figure 5C,F); while a significant iNOS staining was detected in damaged tissues from the ARS group (Figure 5D,F). Treatment with Colomast^®^ significantly reduced iNOS staining (Figure 5E,F).

### 2.7. Effect of Colomast^®^ Pre-Treatment on Nitrotyrosine Formation, Poly (Adp-Ribose) Polymerase (Parp) Activation, Myeloperoxidase Activity (Mpo), and Lipid Peroxidation in Ileum

**Figure 6:**
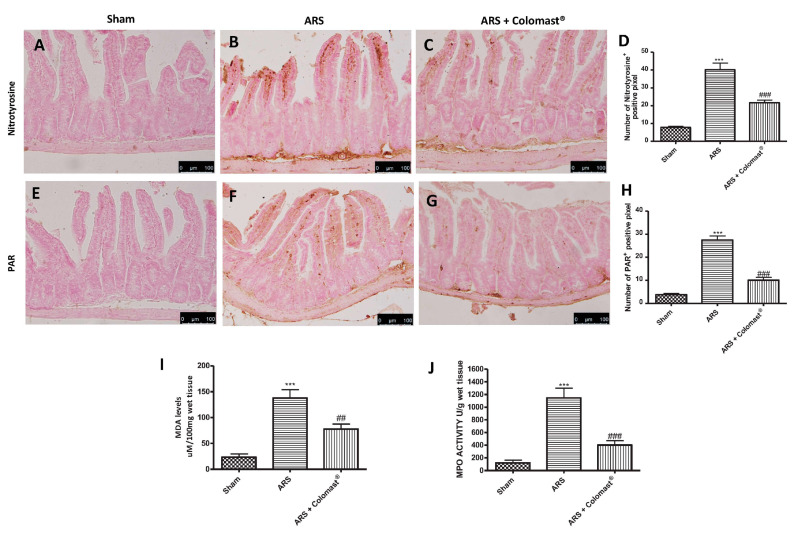
Effect of Colomast^®^ pre-treatment on nitrotyrosine formation, PARP activation, MPO activity, and lipid peroxidation in ileum. Immunohistochemistry evaluation of nitrotyrosine expression: sham (**A**), ARS (**B**), and Colomast^®^ (**C**). Immunohistochemistry evaluation of PARP expression: sham (**D**), ARS (**E**), and Colomast^®^ (**F**). Graphical quantification of nitrotyrosine (**G**) and PARP (**H**) expressions. MDA levels in ileum tissue (**I**). MPO activity in ileum tissue (**J**). Images are figurative of at least three independent experiments. Values shown are means ± SEM of six animals in each group. For the immunohistochemistry, a 20× magnification is shown (100-µm scale bar). *** *p* < 0.001 vs. sham; ## *p* < 0.01 vs. ARS group; ### *p* < 0.001 vs. ARS group.

The expression of nitrotyrosine, a specific indicator of nitrosative stress, and PARP, an indicator of DNA breakdown, was analyzed by immunohistochemical staining: ileum sections from the sham group did not stain for nitrotyrosine and PARP (Figure 6A,D and Figure 6E,H, respectively), whereas sections obtained from the ARS group displayed an important positive nitrotyrosine and PARP immunostaining (Figure 6B,D and Figure 6F,H, respectively) after 2 h of immobilization. Pre-treatment with Colomast^®^ reduced the degree of nitrotyrosine and PARP immunoreactivity in ileum tissue (Figure 6C,D and Figure 6G,H, respectively). Moreover, MDA levels were detected in the ileum tissues as an indicator of lipid peroxidation. A significant increase in MDA levels was observed in immobilized mice compared to sham-treated mice (Figure 6I) and MDA levels were significantly attenuated by Colomast^®^ pre-treatment (Figure 6I). Additionally, neutrophil infiltration was measured through the MPO assay, finding an increased ileal MPO levels in the vehicle group compared to the sham group (Figure 6J). In contrast, administration of Colomast^®^ significantly reduced ileal MPO activity (Figure 6J).

### 2.8. Effect of Colomast^®^ Pre-Treatment on Apoptotic Damage in Ileum Tissue

**Figure 7:**
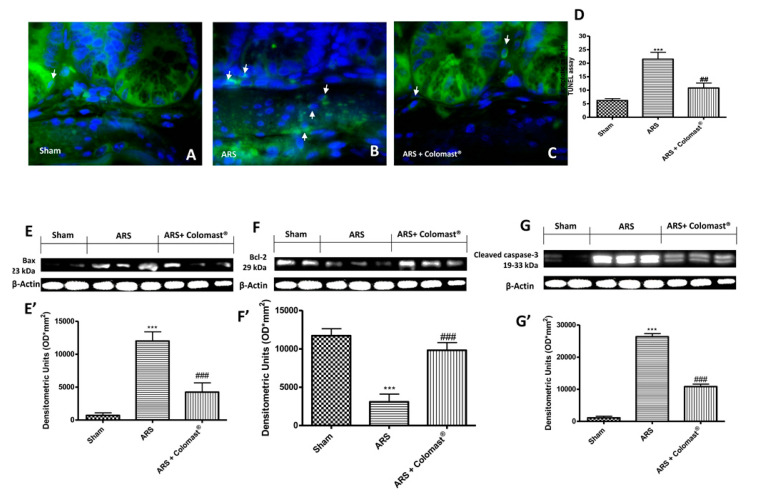
Effects of Colomast^®^ pre-treatment on apoptotic damage. TUNEL staining of ileum sections: sham (**A**); ARS (**B**); Colomast^®^ (**C**); graphical quantification (**D**). Western blots and respectively densitometric analysis of Bax (**E**,**E’**), Bcl-2 (**F**,**F’**), and cleaved-caspase-3 (**G**,**G’**). A demonstrative blot of lysates with a densitometric analysis for all animals is showed. Images are figurative of at least three independent experiments. Values shown are means ± SEM of six animals in each group. For TUNEL staining, a 100× magnification is shown (25-µm scale bar). *** *p* < 0.001 vs. sham; ## *p* < 0.01 vs. ARS group; ### *p* < 0.001 vs. ARS group.

Since Colomast^®^ reduces ARS-induced tissue damage and inflammation, we investigated whether Colomast^®^ plays a role on apoptosis in ileum tissues during restraint stress by terminal deoxynucleotidyl nick-end labeling (TUNEL) assay. As shown in Figure 7A, a low level of TUNEL-positive staining was detected in the sham group, whereas a significant number of TUNEL-positive cells were observed in mice at 2 h after ARS (Figure 7B,D). Administration of Colomast^®^ (20 mg/kg) exerted a significant anti-apoptotic effect (Figure 7C,D). To confirm the anti-apoptotic effect of Colomast^®^, the expression of the pro-apoptotic Bax and the anti-apoptotic Bcl-2 proteins was assessed by Western blot analysis. The Bax expression was appreciably increased in homogenized ileum tissues from mice after 2 h of immobilization compared to the sham group (Figure 7E,E’). Colomast^®^ pre-treatment prevented the ARS-induced Bax expression (Figure 7E,E’). The level of Bcl-2 was significantly decreased in the vehicle group and Colomast^®^ administration was able to enhance Bcl-2 expression at levels similar to the sham (Figure 7F,F’). Moreover, since it is well known that caspases play pivotal roles in apoptosis, we also observed the expression of cleaved-caspase-3 by Western blot analysis. The results obtained showed a significantly increased level of cleaved-caspase 3 in the vehicle group compared to the sham group, while oral treatment with Colomast^®^ reduced markedly the cleaved-caspase-3 expression (Figure 7G,G’).

### 2.9. Effect of Colomast^®^ Pre-Treatment on Tight Junction Expression in Ileum

**Figure 8:**
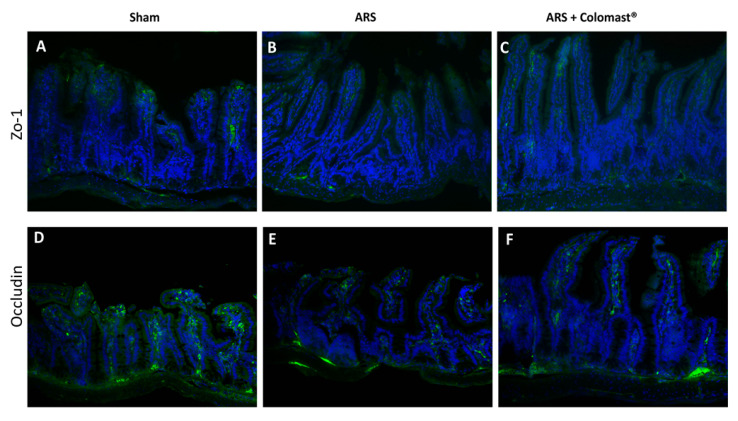
Effect of Colomast^®^ pre-treatment on tight junction expression in ileum. Immunofluorescence in ileum section of ZO-1 in sham (**A**), ARS (**B**), Colomast^®^ (**C**), and Occludin in sham (**D**), ARS (**E**), and Colomast^®^ (**F**). Images are figurative of at least three independent experiments. Values shown are means ± SEM of six animals in each group. For the immunofluorescence, a 20× magnification is shown (100-µm scale bar).

During the inflammatory process, tissue permeability is modified in part by changes in TJs [14,25]. Since ZO-1 and Occludin are implicated in TJ regulation and were used as marker of cellular barrier integrity [26]. Two hours after restraint stress, ileum sections showed a significant immunofluorescence decreased in ZO-1 (Figure 8B) and Occludin (Figure 8E) compared to the sham group (Figure 8A,D, respectively). Oral administration of Colomast^®^ was able to restore the expression of both TJs (Figure 8C,F).

### 2.10. Effect of Colomast^®^ Pre-Treatment on Tj Expression in Brain

**Figure 9:**
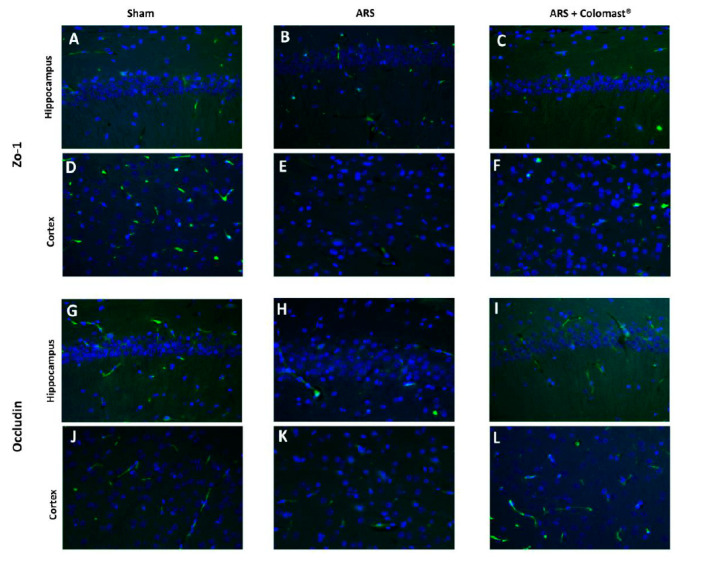
Effect of Colomast^®^ pre-treatment on tight junction expression in the brain. Immunofluorescence of ZO-1 in hippocampus and cortex respectively: sham (**A**,**D**); ARS (**B**,**E**); Colomast^®^ (**C**,**F**). Immunofluorescence of Occludin in hippocampus and cortex respectively: sham (**G**,**J**); ARS (**H**,**K**); Colomast^®^ (**I**,**L**). Images are figurative of at least three independent experiments. Values shown are means ± SEM of six animals in each group. For the immunofluorescence, a 40× magnification is shown (75-µm scale bar).

Since it has been demonstrated that TJ is numerous in the blood brain barrier [27], ARS induced a decrease in hippocampal and cortex expressions of ZO-1 (Figure 9B,E, respectively) compared to sham (Figure 9A,D, respectively), while Colomast^®^ treatment was able to restore the expression of ZO-1 in both hippocampus and cortex (Figure 9C,F, respectively).

At the same way, the expression of Occludin in the vehicle group decreased in hippocampal and cortex sections (Figure 9H,K, respectively) compared to the sham group (Figure 9G,J, respectively). Oral administration of Colomast^®^ restored Occludin expression (Figure 9I,L, respectively) at levels comparable to sham.

### 2.11. Effect of Colomast^®^ Pre-Treatment on Brain Tissue Damage

**Figure 10:**
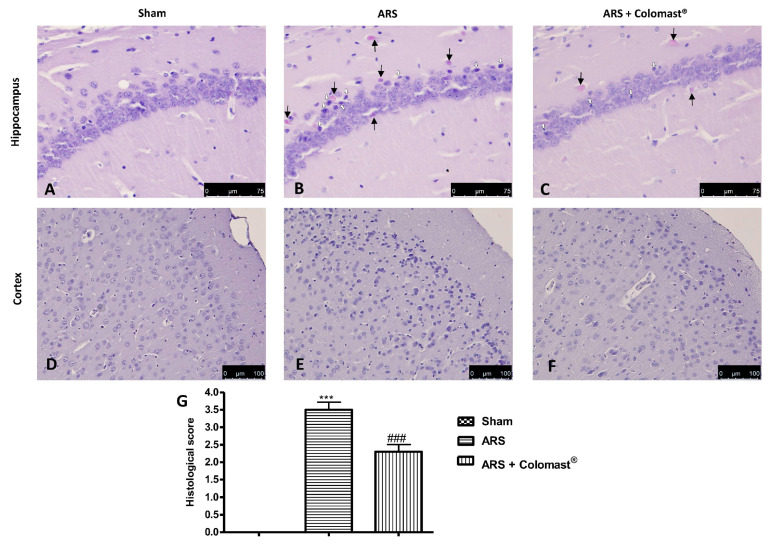
Effect of Colomast^®^ pre-treatment on brain tissue damage. Histological evaluation of hippocampus in sham (**A**), ARS (**B**), Colomast^®^ (**C**), and cortex in sham (**D**); ARS (**E**); Colomast^®^ (**F**); Histological score (**G**). The black and white arrow indicated injured neurons (cell with pyknotic nuclei or acidophilic cytoplasm). Images are figurative of at least three independent experiments. Values shown are means ± SEM of six animals in each group. For the histology, a 40× magnification is shown (75-µm scale bar) for hippocampus, a 20× magnification is shown (100-µm scale bar) for cortex. *** *p* < 0.001 vs. sham; ### *p* < 0.001 vs. ARS group.

To evaluate the severity of the damage in brain tissue after 2 h of immobilization, sections of the hippocampus and cortex obtained from each group were stained with hematoxylin/eosin (H/E). Sections from control mice showed a regular architecture with numerous and closely organized nerve cells in the hippocampal and cortex (Figure 10A,D respectively and Figure 10G). Reduced number of nerve cells, disorganized and degenerate cells were found in the hippocampal and cortex in the stressed-group (Figure 10B,E respectively and Figure 10G) compared to the control group. Colomast^®^ pre-treatment was able to protect against the stress-induced neurodamage (Figure 10C,F respectively and Figure 10G).

### 2.12. Effect of Colomast^®^ Pre-Treatment on Cell Death and Cellular Proliferation in Hippocampus

**Figure 11:**
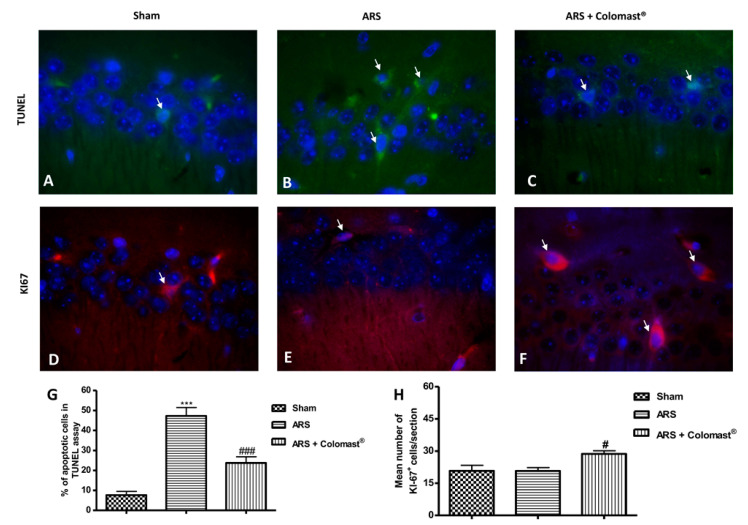
Effect of Colomast^®^ pre-treatment on cell death and cellular proliferation in the hippocampus. TUNEL staining of hippocampus sections: sham (**A**); ARS (**B**); Colomast^®^ (**C**); graphical quantification (**G**). Immunofluorescence of Ki-67 in hippocampus sections: sham (**D**); ARS (**E**); Colomast^®^ (**F**); graphical quantification (**H**). Images are figurative of at least three independent experiments. Values shown are means ± SEM of six animals in each group. For TUNEL staining and immunofluorescence, a 100× magnification is shown (25-µm scale bar). *** *p* < 0.001 vs. sham; # *p* < 0.05 vs. ARS group; ### *p* < 0.001 vs. ARS group.

Apoptosis in the hippocampal area was assessed by TUNEL staining. A low level of TUNEL-positive staining was detected in the sham group (Figure 11A,G), whereas a significant number of TUNEL-positive cells were observed in the ARS group (Figure 11B,G). Colomast^®^ pre-treatment was able to protect against the stress-induced neurodamage, as the percentage of apoptotic neurons declined significantly (Figure 11C,G).

Additionally, Ki-67 expression was studied by immunofluorescence analysis. Ki-67 is a nuclear protein present solely in dividing cells and is therefore used as a marker for cellular proliferation [28]. No significant difference was noted between the sham group and stressed group (Figure 11D,E,H), but the number of actively dividing cells in the sub-granular zone of the dentate gyrus was found to be significantly increased in the Colomast^®^ group (Figure 11F,H) compared to stressed mice indicated by a marked augmentation in the number of Ki-67-positive cells in brain sections from the Colomast^®^ group.

### 2.13. Effect of Colomast^®^ Pre-Treatment on Behavioral Alteration and Sucrose Consumption (%)

**Figure 12:**
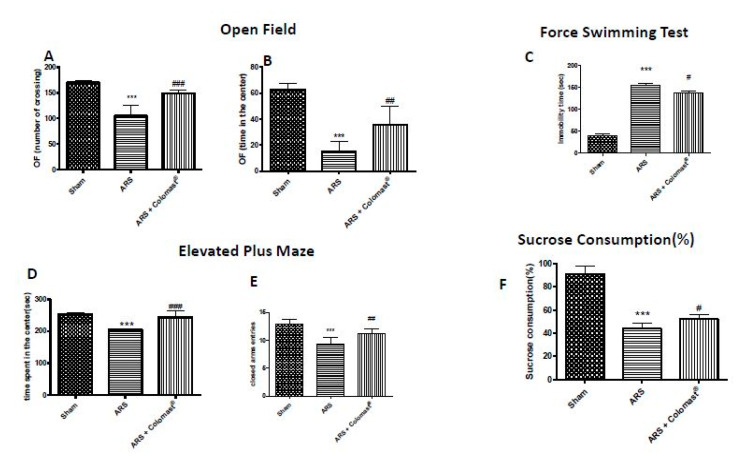
Effect of Colomast^®^ pre-treatment on behavioral alteration and sucrose consumption (%). Open field test: number of crossing (**A**), time spent in the center (**B**). Force swimming test (**C**). Elevated plus maze test: time spent in the center (**D**), number of entries in the open arms (**E**). Sucrose consumption (%) (**F**). Values shown are means ± SEM of six animals in each group. *** *p* < 0.001 vs. sham; # *p* < 0.05 vs. ARS group; ## *p* < 0.01 vs. ARS group; ### *p* < 0.001 vs. ARS group.

The effect of Colomast^®^ on anxiety and depression stress-associated were evaluated using different behavioral tests. Two hours after immobilization, mice significantly decreased the total number of squares crossed compared to the control group (Figure 12A). There was also a decrease in central squares in the ARS group compared to the control group (Figure 12B). Oral treatment with Colomast^®^ increased the number of squares crossed and time spent in central squares compared to the ARS group as clearly demonstrated by the open field (OF) test. Furthermore, 2 h after immobilization, mice exhibited a significant increase of immobility time in the force swimming test (FST) compared to sham group while Colomast^®^-treated mice significantly decreased the immobility period (Figure 12C). The elevated plus-maze (EPM) behavioral test indicated an important decrease in time spent (Figure 12D) and in the number of entries (Figure 12E) in the open arms in ARS-injured mice which was significantly increased with Colomast^®^ pre-treatment. Moreover, we evaluated the sucrose preference test, an important method used to evaluate anhedonia, which is the principal symptom of depression. The sucrose intake was significantly reduced in the vehicle group compared to the control group. On the contrary, the sucrose consumption in mice treated with Colomast^®^ was significantly increased compared to the ARS group (Figure 12F).

## 3. Discussion

Stress has negative effects on health and plays an important role in the predisposition to many physical illnesses [29,30]. Stress can be caused by various factors as biological causes (burns, injuries, surgical operation, high temperature variations, forced immobilization, severe metabolic disorders, etc.), but also psychophysical ones (fear, pain, sadness, etc.) [31]. Age can also be a risk factor for stress [32]. Stress is an important risk factor in the clinical onset and severity of important immunologic disorders [33], including allergy, asthma, and functional and inflammatory gastrointestinal disorders. In addition, stress affects some areas of the brain, particularly the hippocampus [34]. It was previously shown that a homeostatic relationship exists between the GI tract and the central nervous system (CNS) and disturbances to one may rapidly influence the other [35,36]. Animal models demonstrate disturbances to the GI tract mucosa (e.g., inflammation, ischemia) or microbiota (e.g., probiotic supplementation) that acutely influences the expression of key CNS mediators that modulate behavior and cognition [37]. Both emotional and physical stress are one of the major contributory factors that stimulate numerous intracellular pathways, thus leading to increased inflammation and free-radical generation causing oxidative damage [38]. Based on this evidence, understanding the molecular mechanisms can provide a new strategy for clinical prevention and treatment of the stress syndrome.

Colomast^®^ is a novel formulation containing Adelmidrol and sodium hyaluronate. Adelmidrol, a diethanolamide derivative of azelaic acid, belongs to the ALIAmide family that showed great efficacy in the treatment of pain and inflammation comparable to PEA in studies in vivo and in vitro [39,40,41]. As a PEA analogue, the pharmacological properties of Adelmidrol can be related to its ability to down-modulate mast cells activation and mast cells mediator release [42,43]. For this reason, in the last years, Adelmidrol has been considered a successful treatment for inflammatory disease. In addition, our previous studies showed that the combination of Adelmidrol with hyaluronic acid improved the inflammatory signs in an osteoarthritis model [22]. In the same way, the protective effects of the combination with sodium hyaluronate in a cystitis model [16] and in a spinal cord injury model [23] confirmed the anti-inflammatory properties in both an acute and chronic stage [15,19].

In light of the above, we investigated the double effect of Colomast^®^ as a possible treatment for gastrointestinal and brain disorders associated to restraint stress. The first results obtained showed that Colomast^®^ was able to modulate the HPA axis by inhibiting hypothalamic corticotrophin-releasing hormone (CRH) levels, and decreasing plasma concentrations of adrenocorticotrophic hormone (ACTH) and CORT. Several studies reveal that CRH also plays a major role in the regulation of GI functions, such as altering intestinal transit, accelerating colonic motility, and stimulating defecation [44,45]. As a result, in the present study, defecation was significantly activated, reflected by an increased number of fecal pellets. 

One of the most important inflammatory signs that influences ARS is the loss of the intestinal permeability barrier triggered by an alteration in TJs expression [14], which acts as a regulated semi permeable barrier that limits the passive diffusion of solutes across the paracellular pathway between adjacent cells [46,47]. In accordance with previous studies [14,48], we demonstrated that ARS caused an alteration of markers of cellular barrier integrity, such as ZO-1 and Occludin, and that oral administration of Colomast^®^ was able to restore both TJs expression in the ileum of stressed mice. Moreover, our results showed that Colomast^®^ pre-treatment at the dose of 20 mg/kg was able to decrease the tissue damage and the structural abnormalities of the gastrointestinal tract in mice subjected to immobilization. Furthermore, the administration of Colomast^®^ restored the integrity of mucous secreting cells in the GI tract, as demonstrated by alcian blue/PAS staining technique. Later, we focused our attention on the ileum particularly susceptible to immobilization stress. Additionally, considering the previously capacity of Adelmidrol to inhibit mast cells degranulation [15] and considering that mast cells are able to release different pro-inflammatory mediators and thus increase inflammation of leukocytes in inflammatory states, we clarified the role of Colomast^®^ on these pointers. Our results confirmed that Colomast^®^ was able to counteract mast cells degranulation in the ileum and attenuate polymorphonuclear cells infiltration, as demonstrated by the reduction of MPO activity, a marker of neutrophilic infiltration.

Furthermore, the protective effects of this new formulation may be attributable, in part, to suppression of the inflammatory response via down-regulation of the NF-κB pathway. Since NF-κB is involved in the gene regulation of various inflammatory proteins and mediators such as TNF-α, IL-1β, and iNOS, we have evaluated the effect of Colomast^®^ on all these expressions. Oral administration of Colomast^®^ significantly reduced the nuclear translocation of NF-κB in ileum tissues as well as reduced up-regulation of cytokines. On the other hand, Colomast^®^ was able to increase IkB-α expression. These results are in agreement with previous studies in which the oral treatment with Adelmidrol modulated the NF-κB pathway and pro-inflammatory cytokines release [15,41]. Activated neutrophils produce ROS and reactive nitrogen species within intestinal mucosa, which provoke oxidative stress, leading to DNA single-strand damage, PAR synthetase activation, and lipid peroxidation [49,50]. In accord with this process, we detected an increased positive staining for nitrotyrosine and PAR in mice subjected to immobilization, which was blunted by Colomast^®^ pre-treatment, as well as decreased MDA formation. In the sequence of events that accompany the inflammatory process, was also saw the involvement of the apoptotic pathway in experimental models of restraint stress in vivo [51,52]. Our results suggest that Colomast^®^ prevents the loss of the anti-apoptotic pathway and, at the same way, reduces the activation of the pro-apoptotic pathway. These data are confirmed by TUNEL staining which showed a reduction of number of TUNEL-positive cells in the ileum section after Colomast^®^ administration.

Another important site rich in TJs is the blood brain barrier (BBB) which is in charge of (i) regulating the passive diffusion of polar constituents from the blood to the brain, (ii) arbitrating the passage of nutrients to the brain parenchyma as well as the efflux from the brain of toxic metabolites and xenobiotics, (iii) controlling the migration of circulating immune cells [53,54]. In our study, we demonstrate that Colomast^®^ pre-treatment at the dose of 20 mg/kg is able to restore TJs expression not only in the ileum section but also in the brain section, in particular, in the hippocampus and cortex regions. Alteration of TJ resulted in a histological damage of the hippocampus and cortex in the mice subjected to immobilization, while treatment with Colomast^®^ restored the normal architecture of the brain to levels similar to the control group. 

The literature indicates that the number of damaged and apoptotic neurons increased in response to stress [51]. At the same time, another important effect of restraint stress is its suppressing influence on neurogenesis in the hippocampus, often measured using Ki-67, a mitotic biomarker [55,56].

This is in line with our results, in fact, we observed that Colomast^®^ was able to decrease death cells not only in the ileum but also in the brain, in particular in the hippocampal zone, as demonstrated by a lower number of apoptotic cells compared to vehicle group in TUNEL staining. On the contrary, we demonstrated that Colomast^®^ promotes cell proliferation, as can be seen from the increase of the KI-67 signal. Finally, BBB alteration leads to a series of depressive and anxiety-like symptoms that were significantly inhibited by Colomast^®^ treatment as demonstrated by behavior tests. Indeed, over the last two decades, several studies have demonstrated that the immune system dysfunction and inflammation with the systemic increase of pro-inflammatory cytokines have a central role in the pathophysiology of major depression, critically contributing to treatment-resistance [57,58]. Therefore, we can suggest that Colomast^®^ can revert depression and anxiety-related behavior in the model of ARS due to its anti-inflammatory properties.

In conclusion, our data clearly demonstrate that Colomast^®^, a new formulation of Adelmidrol and sodium hyaluronate, at a dose of 20 mg/kg, reduces overexpression of stress hormones, TJ alteration, and the inflammatory process, and modulates the apoptosis pathway in an experimental model of ARS in vivo. These results suggest that Colomast^®^ could be a useful pharmacological strategy for treating ARS and its comorbidities.

## 4. Materials and Methods

### 4.1. Animals

Adult CD1 mice (8–9 week old male; 25–30 g; Envigo, Milan, Italy) were housed in a controlled environment and provided with standard rodent chow and water ad libitum. The University of Messina Review Board approved the research and the animal care was in conformity with current legislation for the protection of animals (Directive 2010/63/EU; protocol number No. 650/2017-PR dated 21/08/2017).

### 4.2. Colomast^®^ Composition and Measurement of Adelmidrol Plasma Absorption Rate by Lc-Ms/Ms (Preliminary Data)

The preliminary experiment was a performed measurement of the Adelmidrol serum absorption rate. Colomast^®^ is a new formulation of Adelmidrol and sodium hyaluronate. For oral administration, Colomast^®^ was formulated in pellet with 15% glyceryl dibehenate, a waxy material that can be used as a matrix-forming excipient for drug sustained release. Colomast^®^ pellet 20 mg/kg was administered to mice (*n* = 5) in carboxymethylcellulose (CMC) 2.5% by oral gavage and blood samples collection was performed at the beginning of experimentation and after 30 min, 3, 6, and 24 h. Plasma samples preparation was performed as previously described [59]. Briefly, 100 µL of plasma samples were diluted in 900 µL of acetonitrile and the absorption rate of Adelmidrol in mice plasma was measured using a LC-MS/MS (Agilent Technologies G6470A) as a function of time. The stock solution of Adelmidrol was prepared in methanol and the five point calibration curve was prepared by dilution in acetonitrile from the stock solution as previously described [60].

### 4.3. Animal Model of Restraint Stress

In other set of animals, immobilization stress was induced in mice by fixation for 2 h of the four extremities with an adhesive tape under brief ether anesthesia. Since fecal pellet output is known to increase under stress conditions, the stools were collected to document the stress effect during the 2 h stress period. Mice were euthanized after a 2 h observation period [14].

### 4.4. Experimental Groups

Mice were randomly allocated into the following groups (*n* = 12):

- ARS + Veh: mice were immobilized as described above and vehicle CMC 2.5% was administered;
- ARS + Colomast^®^: mice were immobilized and Colomast^®^ (20 mg/kg) in CMC 2.5% was administered by oral gavage 30 min before the immobilization.
- Sham + Veh: mice were only briefly anesthetized with isoflurane and thereafter allowed to move freely in their cages over the following 2 h.
- Sham + Colomast^®^: the same conditions of Sham+Veh group, but Colomast^®^ (20 mg/kg) in CMC 2.5% was administered by oral gavage (data not shown).

The doses of Colomast^®^ were chosen based on a dose-response study carried out in our lab (data not shown).

At the end of the experiment, animals were sacrificed under anesthesia and stomach, ileum, colon, and brain were collected and fixed in 10% neutral buffered formalin and embedded in paraffin for both histological and immunohistochemical examinations or stored at −80 °C for further analyses.

### 4.5. Measurement of Hormones Stress

After 2 h of immobilization, plasma was collected and frozen in −80 °C. The levels of CRH, ACTH CORT were measured in plasma as previously described [61].

### 4.6. Histological Examination

Tissue sections (7 μm) were deparaffinized, stained with hematoxylin/eosin (H/E) and studied by using light microscopy connected to an imaging system (AxioVision; Zeiss, Milan, Italy). The histological score was determined as previously described [62,63,64,65].

### 4.7. Alcian Blue/PAS Staining

Alcian blue/PAS staining technique was used to detect acid muco-substances and acetic mucins in different mucous secreting cells of the stomach, ileum and colon. Sections stained with alcian blue/PAS were analyzed using a light microscopy connected to an imaging system (AxioVision; Zeiss, Milan, Italy) as previously described [14].

### 4.8. Staining of Mast Cells

Two hours after immobilization, for identification of mast cells, ileum and colon tissue sections were stained with toluidine blue as described previously [66,67].

### 4.9. Myeloperoxidase Activity

Myeloperoxidase (MPO) activity, an index of polymorphonuclear cell accumulation, was determined in the ileum tissue as previously described [68].

### 4.10. Western Blot Analysis of Cytosolic and Nuclear Extracts from Ileum Tissue

Cytosolic and nuclear extracts were prepared as previously described [69,70]. The following primary antibodies were used for cytosolic fraction: anti-IκBα (1:500, Santa Cruz Biotechnology, #sc1643), anti-Bax (1:500, Santa Cruz Biotechnology, Dallas, TX, USA, #sc7480), anti-Bcl-2 (1:500, Santa Cruz Biotechnology, #sc7382), cleaved-caspase 3 (1:500, Cell Signaling, Danvers, MA, USA), and β-actin (1:1.000; Santa Cruz Biotechnology, #sc8432). The following primary antibodies were used for nuclear fraction: anti-NF-κB p65 (1:500, Santa Cruz Biotechnology, #sc8008), and lamin A/C antibody (1:5000; Sigma-Aldrich, St. Louis, MO, USA). Protein expression was quantified by densitometry with BIORAD ChemiDocTM XRS+software and standardized to β-actin and lamin A/C levels [71].

### 4.11. Immunohistochemical Localization of iNOS, Nitrotyrosine, Poly (ADP-ribose) Polymerase (PARP)

Immunohistochemical analysis was performed as previously described [41,72]. The ileum tissue sections were incubated overnight with anti-iNOS (1:250, BD-transduction), anti-nitrotyrosine (1:250, Merck-Millipore, Burlington, MA, USA), anti-PARP (1:200, Santa Cruz Biotechnology, #sc1561) antibodies.

Images were collected using a Zeiss microscope (Carl Ziess, Oberkochen, Germany) and Axio Vision software (Carl Zeiss). The digital images were analyzed in ImageJ (National Institutes of Health, Bethesda, MD, USA) using the color deconvolution plug-in. When the Immunohistochemistry Profiler plugin is selected, it mechanically plots a histogram profile of the deconvoluted diaminobenzidine image, and a corresponding scoring log is exhibited [73]. The histogram profile refers to the positive pixel intensity value obtained from a computer program [74].

### 4.12. Measurement of Cytokines

Tumor necrosis factor alpha (TNF-α) and interleukin (IL)-1β levels were evaluated in the ileum tissues after 2 h of the restraint period as previously described [75].

### 4.13. Thiobarbituric Acid-Reactant Substances Measurement (MDA Levels)

Thiobarbituric acid-reactant substances measurement, which is considered a good indicator of lipid peroxidation, was determined in the ileum tissue at 2 h after immobilization as previously described [76].

### 4.14. Terminal Deoxynucleotidyl Nick-End Labeling (TUNEL) Assay

TUNEL staining for apoptotic cell nuclei and DAPI staining for all cell nuclei were performed in ileum and brain sections as described previously [14,51,77,78]. The index of apoptosis was expressed as the number of positively stained apoptotic cells/the total number of cells counted ×100%.

### 4.15. Immunofluorescence Localization of ZO-1, Occludin, KI-67

Sections were processed for immunofluorescence staining as previously described [79]. Sections were incubated with anti-ZO-1 (1:500; Invitrogen, Carlsbad, CA, USA), anti-Occludin (1:500, Invitrogen), anti-KI-67 (1:500; Abcam) antibodies. Each picture was digitalized and analyzed as previously described [79].

### 4.16. Behavioral Testing

Behavioral assessments on each mouse were made 2 h after immobilization. Open field (OF) test, elevated plus-maze (EPM) test, force swimming test (FST) were executed as previously explained [80,81].

### 4.17. Sucrose Consumption (%)

The sucrose preference test was conducted as previously described [82].

### 4.18. Materials

Colomast^®^ were obtained from Epitech Group SpA (Saccolongo, Italy). Unless otherwise stated, all compounds except where differently specified, were purchased from Sigma-Aldrich Company Ltd. (St. Louis, MO, USA).

### 4.19. Statistical Evaluation

All values are expressed as mean ± standard error of the mean (SEM.) of N observations. The figures shown are representative of the least 3 experiments performed on diverse experimental days on tissue sections collected from all animals in each group. For in vivo studies, N represents the number of animals used. The results were analyzed by one-way ANOVA followed by a Bonferroni post-hoc test for multiple comparisons. A P value less than 0.05 was considered significant.

## Figures and Tables

**Table 1:** In vivo Adelmidrol absorption**.** Adelmidrol absorption: all serum levels of Adelmidrol were less than limit of quantification (LOQ).

Mouse	Time	LOQ (ng/mL)
1	T0	<71.4
	30 min	<71.4
	T3	<71.4
	T6	<71.4
	T24	<71.4
2	T0	<71.4
	30 min	<71.4
	T3	<71.4
	T6	<71.4
	T24	<71.4
3	T0	<71.4
	30 min	<71.4
	T3	<71.4
	T6	<71.4
	T24	<71.4
4	T0	<71.4
	30 min	<71.4
	T3	<71.4
	T6	<71.4
	T24	<71.4
5	T0	<71.4
	30 min	<71.4
	T3	<71.4
	T6	<71.4
	T24	<71.4

## Abbreviations

ACTH	adrenocorticotrophic hormone
ALIAmide	autacoid local injury antagonist amide
ARS	acute restraint stress
BBB	blood brain barrier
CNS	central nervous system
CORT	corticosterone
CRH	hypothalamic corticotrophin-releasing hormone
EPM	elevated plus-maze
FST	force swimming test
GI	gastrointestinal
HPA	hypothalamic-pituitary-adrenal axis
IL-1β	interleukin-1β
MPO	myeloperoxidase
OF	open field
PARP	poly (ADP-ribose) polymerase
PEA	palmitoylethanolamide
TJ	tight junction
TNF-α	tumor necrosis factor alpha
TUNEL	terminal deoxynucleotidyl transferase dUTP nick end labeling assay
ZO-1	zonula occludens-1 (ZO-1
